# Molybdenum Disulphide Precipitation in Jet Reactors: Introduction of Kinetics Model for Computational Fluid Dynamics Calculations

**DOI:** 10.3390/molecules27123943

**Published:** 2022-06-20

**Authors:** Michał Wojtalik, Krzysztof Wojtas, Weronika Gołębiowska, Maria Jarząbek, Wojciech Orciuch, Łukasz Makowski

**Affiliations:** Warsaw University of Technology, Faculty of Chemical and Process Engineering, Warynskiego 1, 00-645 Warsaw, Poland; michal.wojtalik.dokt@pw.edu.pl (M.W.); krzysztof.wojtas@pw.edu.pl (K.W.); weronika.golebiowska.stud@pw.edu.pl (W.G.); maria.jarzabek.stud@pw.edu.pl (M.J.); wojciech.orciuch@pw.edu.pl (W.O.)

**Keywords:** molybdenum disulphide, nanoparticle, precipitation, jet reactor, kinetics, computational fluid dynamics

## Abstract

In our previous work, we used the population balance method to develop a molybdenum disulphide kinetics model consisting of a set of differential equations and constants formulated to express the kinetics of complex chemical reactions leading to molybdenum disulphide precipitation. The purpose of the study is to improved the model to describe the occurring phenomena more thoroughly and have introduced computational fluid dynamics (CFD) modelling to conduct calculations for various reactor geometries. CFD simulations supplemented with our nucleation and growth kinetics model can predict the impact of mixing conditions on particle size with good accuracy. This introduces another engineering tool for designing efficient chemical reactors.

## 1. Introduction

The main goals of chemical and process engineering are to select the appropriate apparatus and process operating parameters. The designer can efficiently combine time and space scale analyses to determine the proper configuration. Time scale analysis determines how accurate mathematical models one should use for the process description, thereby allowing the designer to choose from classical chemical reactor engineering methods, all the way to the most advanced direct models for all the process stages and then to select a mathematical modelling method based on mass, momentum, energy, and population balances. Numerous works on time scale analysis [1,2] have shown that the course of many processes of practical importance can be influenced by choosing the reactant contact method, the mixing intensity (including the choice of local flow and mixing parameters such as the local shear rate, stresses, and dissipation rate), and the process running method (e.g., continuous, semiperiodic, and periodic). Proper selection of process methods and parameters often results in better product yield and quality and fewer byproducts (including environmentally harmful ones) and often reduces the number of production stages required. Recently, computational models in combination with computational fluid dynamics (CFD) have been increasingly used for this purpose [3,4,5,6,7,8].

CFD was pioneered by Johnson and Richardson [9], who developed an iterative numerical method of calculating Laplace equations to determine variables based on the numbers calculated in the previous model iteration. Because all the numbers were calculated manually, the method applicability was originally considerably limited. The introduction of computers in the early 1950s led to a major development in computational fluid mechanics methods. The first works were related to the aviation, automotive, and armaments industries [10]; it was not until the 1980s that CFD was first applied to chemical engineering [11]. Since then, many scientific papers have used this method to control the final characteristics of chemical products by selecting the process conditions, particularly the reactant mixing conditions [3,12,13,14,15].

Molybdenum disulphide (MoS_2_) is a transition-metal dichalcogenide (TMD) and a valuable 2D-nanomaterial used in a wide range of industrial applications. The main reason for such a wide range of applications of molybdenum disulphide in various fields is its 2D terrace structure and its crystalline properties. One promising application is the use of MoS_2_ in several catalytic reactions such as hydrogen evolution reactions (HERs), hydrodesulphurization, oxygen reduction reactions (ORRs), and methane conversion [16,17,18,19,20]. In addition, MoS_2_ has been used for many years as a dry lubricant and an oil additive. New research also shows the possibility of improving MoS_2_ dispersion by synthesizing MoS_2_ particles on the surface of graphene materials [21]. These applications could be essential in future environmental projects involving sustainable energy sources, especially because the environmental impact of wet chemical synthesis is acceptable [22]. Owing to the many possible MoS_2_ applications, the MoS_2_ synthesis reaction kinetics must be accurately described, and the impact of the process conditions on the final product must be accurately modelled [23]. Most of the modern applications of molybdenum disulphide require a chemically pure material with reproducible properties. This wide range of applications makes molybdenum disulphide one of the most frequently studied materials in terms of its synthesis and properties. Particle size is of particular importance for catalysts and lubricants, but it is also important for applications to improve the properties of polymeric materials due to the necessity of achieving suitable dispersions. Recrystallisation or other processes related to the processing of the raw material are more commonly used in the production of electronic materials, but, even then, a substance with a known particle size distribution and morphology is essential. This type of material can be obtained by precipitation, also known as reactive crystallisation, in which the driving force behind the process is the supersaturation resulting from a chemical reaction.

To date, no studies modelling the effects of the reactor geometry and flow conditions on the precipitation of MoS_2_ particles have previously been published. Therefore, this study presents the first such attempt and draws conclusions from the obtained results. The study findings shed new light on MoS_2_ precipitated using wet chemical synthesis, which is particularly important for optimally designing reactor geometries to carry out the described process. This will be crucial for the application of this method to produce nanoparticles with the desired properties for industrial application.

## 2. Materials and Methods

**Figure 1:**
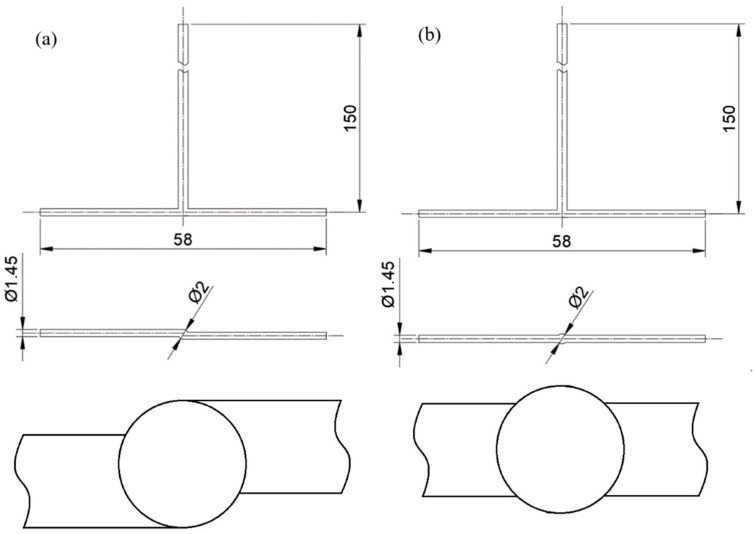
Geometries of (**a**) tangential inlets V-type and (**b**) coaxial inlets T-type impinging jet reactors (all dimensions are given in mm).

The reaction model presented herein is based on our previous work [24]. The model describes the wet chemical synthesis of MoS_2_ in impinging jet reactors (Figure 1), using ammonium heptamolybdate (HMA) and ammonium sulphide (AS) as reaction substrates in citric acid (CA), which acts as a catalyst and allows the reaction pH to be set. To enable reproducibility of the test, information on substrates and their preparation is described in the publications [24,25,26]. It should be noted that the aqueous solution of ammonium sulphide is unstable and degrades in contact with air, eventually leading to precipitation of free sulphur, which can completely distort the results, therefore clean and, above all, properly stored ammonium sulphide should always be used for the process. The model predicts only the particle nucleation and growth kinetics and does not allow for particle aggregation and agglomeration, which also occur extensively during the process. The reaction and particle morphology analysis were described in more detail in previous works [24,25,26]. Previously published experimental data were also used to validate the model [24,25].

Although the sequence of chemical reactions leading to the product has been presented previously [24,25,26], the last reaction given in the sequence was split into two (reaction Equations (8) and (9)). This is a more suitable way to describe the reaction mechanism separating dissociation from precipitation. Therefore, a modified reaction mechanism is given below, which does not affect the computational results or the modelling approach, but only shows a more likely course of the process.

(1) $${\left({\mathrm{NH}}_{4}\right)}_{6}{\mathrm{Mo}}_{7}O_{24}⇆{\left[{\mathrm{Mo}}_{7}O_{24}\right]}^{6-}+6{\left[{\mathrm{NH}}_{4}\right]}^{+},$$

(2) $${\left[{\mathrm{Mo}}_{7}O_{24}\right]}^{6-}+4H_{2}O⇆7{\left[{\mathrm{MoO}}_{4}\right]}^{2-}+8H^{+},$$

(3) $${\left({\mathrm{NH}}_{4}\right)}_{2}S⇆\left[{\mathrm{NH}}_{4}\right]{\mathrm{HS}+\mathrm{NH}}_{3},$$

(4) $$\left[{\mathrm{NH}}_{4}\right]\mathrm{HS}⇆H_{2}{S+\mathrm{NH}}_{3},$$

(5) $$H^{+}+{\mathrm{NH}}_{3}⇆\;{\left[{\mathrm{NH}}_{4}\right]}^{+},$$

(6) $$H_{2}S\to H^{+}{+\mathrm{HS}}^{-},$$

(7) $${\mathrm{HS}}^{-}\to H^{+}{+S}^{2-},$$

(8) $${\left[{\mathrm{MoO}}_{4}\right]}^{2-}+8H^{+}\to {\mathrm{Mo}}^{6+}+4H_{2}O,$$

(9) $${\mathrm{Mo}}^{6+}+3S^{2-}\to {\;\mathrm{MoS}}_{2}↓+\;S↓,$$

(10) $${\mathrm{Mo}}^{6+}+2e^{-}\to {\mathrm{Mo}}^{4+},$$

(11) $$S^{2-}\to S↓+2e^{-}.$$

**Figure 2:**
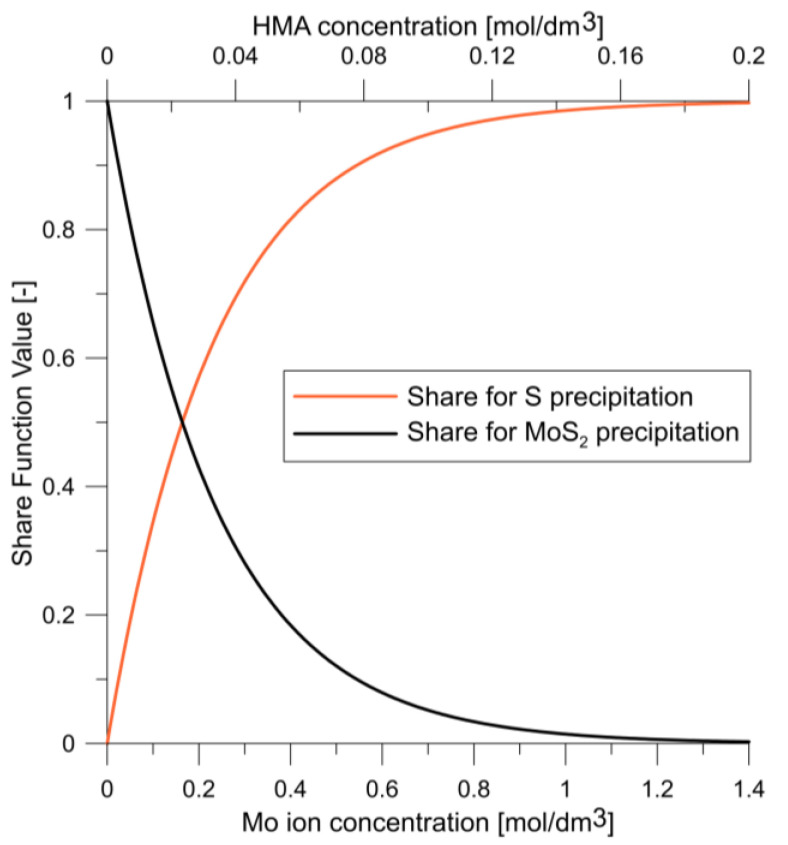
Share function values for a given model.

Under ideal mixing conditions, the model given in Ref. [24] sufficiently described the process. However, in finite volume modelling, the model had to be modified by adding more equations because the original model inaccurately described phenomena at low molybdenum-ion concentrations. The process was previously described as being limited by the concentration of sulphide ions because free sulfur must precipitate to reduce molybdenum from the VI to the IV oxidation state. However, at low molybdenum-ion concentrations, the process is no longer limited solely by the sulphide ion concentration. The concentration of molybdenum ions in the VI oxidation state then becomes essential to the process, which requires two precipitation models (Equations (2) and (3) in Table 1) combined using share functions depending on the molybdenum concentration (Equations (6) and (7) in Table 1) to obtain an accurate mathematical description of MoS_2_ precipitation. The share functions for the HMA concentration are shown in Figure 2.

The model constants were fitted based on the experimental data described in Ref. [24] using nonlinear programming algorithms described in the same publication and assuming ideal mixing conditions in a plug-flow reactor. A similar method of determining the model constants was proposed by Al-Tarazi et al. [27,28]. The model constants are listed in Table 2. The resulting model was further implemented in the CFD code using a user-defined function (UDF) in Ansys Fluent software.

## 3. CFD Modeling

Ansys Fluent 2020R1 software was used to solve steady-state system transport equations in 3D geometry model since it is impossible to model the V-type reactor directly using two-dimensional domain. Calculations were performed based on steady-state conditions without using the micromixing closure model. To predict the chemical reaction course, the flow must initially be calculated in the computing domain. Therefore, due to transitional conditions of the flow in impinging jet reactors [12,13], the shear stress transport (SST) *k*–*ω* turbulence model was employed, and the diffusivity was defined as follows:

(12) $$D=\rho \cdot D_{m}+\frac{\mu_{T}}{Sc_{T}},$$

where $\mu_{T}$ is the turbulent viscosity, $Sc_{T}$ is the turbulent Schmidt number, and the molecular diffusivity is constant:

(13) $$D_{m}=10^{-9}\;\frac{m^{2}}{s}.$$

The SST *k*–*ω* turbulence model contains two transport equations. Because the standard *k*–*ω* and modified *k*–*ε* models are used near walls and in free flow, respectively, the SST *k*–*ω* model performs well for strong pressure gradients [29]. Both models are combined using a mixing function. As described by Bardina et al. [29], the original *k*–*ω* and transformed *k*–*ε* models are multiplied by the *F*_1_ and $1-F_{1}$ mixing functions, respectively, and the equations from both models are summed. *F*_1_ = 1 near the wall and 0 elsewhere in the region.

Turbulent viscosity is a function of the turbulent kinetic energy (*k*) and the specific energy dispersion rate (*ω*). To better estimate the boundary layer detachment, it is further modified using an auxiliary function defined as follows:

(14) $$\mu_{t}=\frac{\frac{\rho k}{\omega }}{\max\left[1;\frac{\Omega F_{2}}{\alpha_{1}\omega }\right]}.$$

For turbulent kinetic energy, the transport equation is given as follows:

(15) $$\frac{\partial \rho k}{\partial t}+\frac{\partial }{\partial x_{j}}\left(\rho u_{j}k-\left(\mu +\sigma_{k}\mu_{t}\right)\frac{\partial k}{\partial x_{j}}\right)=\tau_{tij}S_{ij}-\beta^{*}\rho \omega k.$$

For an energy dissipation rate, the transport equation is as follows:

(16) $$\frac{\partial \rho \omega }{\partial t}+\frac{\partial }{\partial x_{j}}\left(\rho u_{j}\omega -\left(\mu +\sigma_{\omega }\mu_{t}\right)\frac{\partial \omega }{\partial x_{j}}\right)=P_{\omega }-\beta \rho \omega^{2}+2\left(1-F_{1}\right)\;\frac{\rho \sigma_{\omega 2}}{\omega }\;\frac{\partial k}{\partial x_{j}}\;\frac{\partial \omega }{\partial x_{j}}.$$

Model constants $\alpha_{1}$, $\beta^{*}$, κ, $\Phi_{k1}$, $\Phi_{\omega 1}$, $\beta_{1}$, $\gamma_{1}$, $\Phi_{k2}$, $\Phi_{\omega 2}$, $\beta_{2}$, and $\gamma_{2}$ can be found in Ref. [29]. The boundary conditions are the same as those for the standard *k*–*ω* model.

The standard method of moments (SMM) was used to solve the population balance. The basic population balance can be formulated as follows:

(17) $$\frac{\partial f\left(\vec{x},L,t\right)}{\partial t}+\overset{3}{\underset{i=1}{\sum }}\frac{\partial \left[u_{pi}\left(\vec{x},t\right)\;f\left(\vec{x},L,t\right)\right]}{\partial x_{i}}+\frac{\partial \left[G\left(\vec{x},L,t\right),f\left(\vec{x},L,t\right)\right]}{\partial L}\;=B\left(\vec{x},L,t\right)-D\left(\vec{x},L,t\right)\;\;\;\;for\;n=0,\;1,\;2,\;\ldots$$

Using the moments of the number distribution of a crystal size and the equations of nucleation kinetics and crystal growth, the following balance equations for averaged moments can be formulated:

(18) $$\frac{\partial \langle m_{n}\rangle }{\partial t}+\langle u_{pi}\rangle \frac{\partial \langle m_{n}\rangle }{\partial x_{i}}+\langle m_{n}\rangle \frac{\partial \langle u_{pi}\rangle }{\partial x_{i}}\;\;\;\;\;\;\;\;\;\;\;\;\;\;\;\;\;\;\;\;\;\;=\frac{\partial }{\partial x_{i}}\left[D_{pT}\frac{\partial \langle m_{n}\rangle }{\partial x_{i}}\right]+0^{n}\langle R_{N}\rangle +n\langle Gm_{n-1}\rangle +\langle B_{n}\rangle -\langle D_{n}\rangle \;\;\;for\;n=0,\;1,\;2,\;\ldots$$

where the equations for the average values of nucleation rate $R_{N}$, growth rate G, and the considered moments $m_{n}$ are given in Table 1 (Equations (8), (9), and (13)–(15), respectively) and $B_{n}=D_{n}=0$. The total average value for nucleation and growth are combinations of two kinetics equations, as described in Table 1 (Equations (8) and (9)).

To simultaneously consider the effects of the mixture viscosity and density on the flow and the influence of the flow on the precipitation course, the CA concentration was assumed to most strongly influence the fluid properties. Therefore, the parameters entirely depend on the CA concentration. Because the particle concentration never exceeds 3% vol. in the suspension, the particle quantity negligibly affects the mixture rheology [30]. The particle volumetric concentration is represented by the third particle-decomposition moment described in Section 4, and the particles synthesized from the reaction are amorphous composites composed of sulfur and MoS_2_ and, therefore, do not exhibit typical MoS_2_ lubricating properties. The mixture viscosity was calculated using Kendall and Monroe’s formula as follows:

(19) $$\mu_{m}^{\frac{1}{3}}=x_{1}\mu_{1}^{\frac{1}{3}}+x_{2}\mu_{2}^{\frac{1}{3}}.$$

The first compound in the formula is responsible for the CA viscosity, while the second represents the remaining solution—which viscosity was assumed constant—independent of concentration and the same as for water. Using the same principle, the mixture density was calculated. The CA viscosity and density data were originally described in [31] and are listed in Table 3.

**Figure 3:**
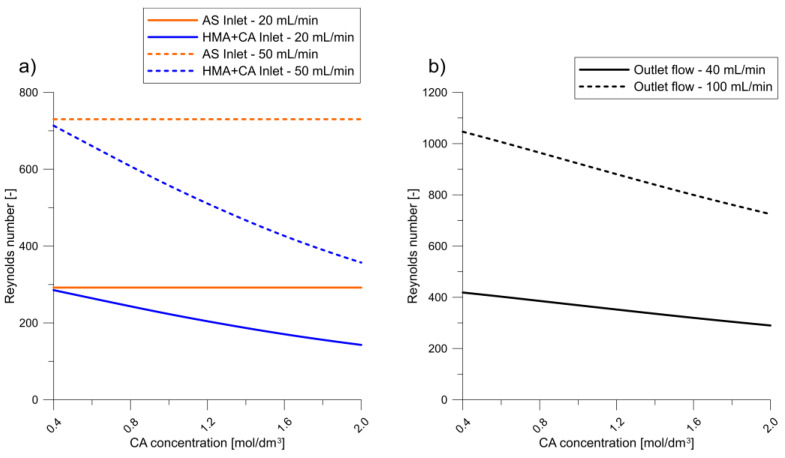
Reynolds numbers at the (**a**) inlets and (**b**) outlet.

Using the determined mixture physicochemical parameters, Reynolds number values were calculated for the reactor inlet and outlet, as shown in Figure 3. Although the reactor inlet and outlet flow are both in the laminar regime, violent collisions and changes in the flow direction cause considerable turbulence in the reaction zone [13]. Therefore, a proper turbulence model must be developed to model the flow profile.

**Figure 4:**
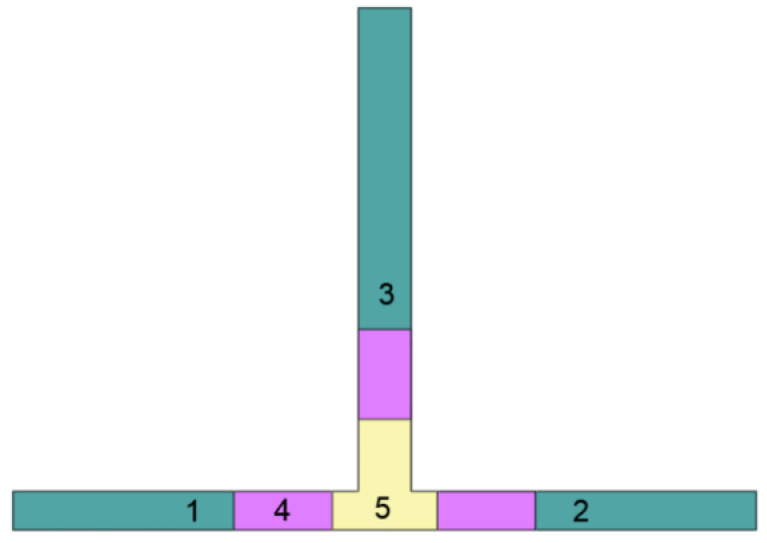
Domain divided into zones: (1) inlet zone of HMA and CA mixture, (2) inlet zone of AS solution, (3) reactor outlet zone, (4) transition zone between zones 1–3 and reaction zone, and (5) reaction zone.

As shown in Figure 4, the computational domain was divided into the following zones to achieve the correct time scale: (1) inlet zone of HMA and CA mixture, (2) inlet zone of AS solution, (3) reactor outlet zone, (4) transition zone between zones 1–3 and reaction zone, and (5) reaction zone. Except for the inlets, which both exhibited the same mesh, each zone was characterized by a higher mesh density than the previous one. The reaction zone was filled with poly-hexcore mesh. The mesh parameters used for both computational domains are listed in Table 4.

**Figure 5:**
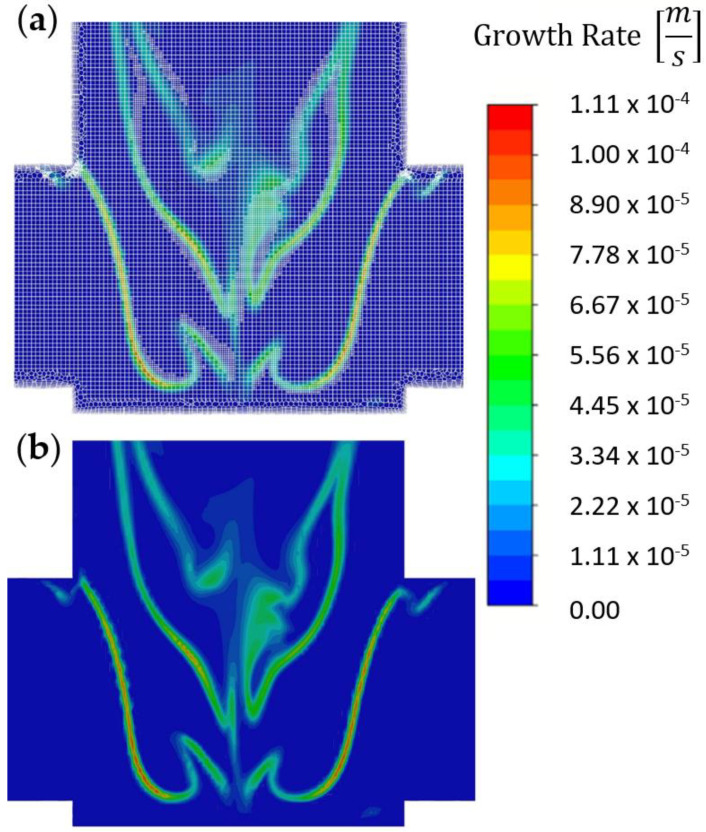
(**a**) Growth rate obtained using adapted mesh and (**b**) growth rate for comparison [m/s].

The mesh of average cell size of 0.02 mm was used in the impingement zone, and the y+ values at the walls in the reaction zone were around 0.1. It was tested that such a grid size was sufficient to obtain mesh independence, i.e., there was no difference in the average values of turbulence quantities (dissipation rate, kinetic energy) in the mixing zone for this and higher mesh densities. However, to reduce the influence of large scalar gradients on the computational results, the grid in the reaction zone was further refined—the particle growth rate was used as the adaptation criterion. An example of the adaptation results is shown in Figure 5.

**Figure 6:**
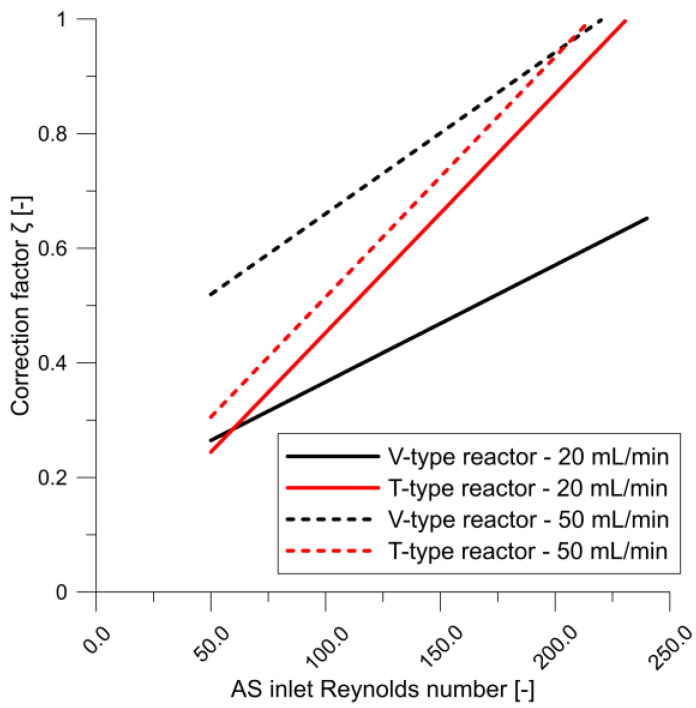
Correction factor (*ζ*) plotted as functions of AS inlet Reynolds number for different reactor types and flow rates.

Because the nucleation and growth model constants were chosen assuming ideal reactor mixing conditions [24], the model had to be corrected to improve the prediction accuracy. Because ideal mixing conditions do not occur in real-life reactors, the constants do not fully reflect the phenomena occurring therein. Therefore, an additional function was introduced to correct the growth rate based on the local Reynolds number. We found that under certain flow conditions, the ideal mixing model predicts MoS_2_ particle sizes very well for higher Reynolds numbers. Those conditions were assumed to be close to ideal mixing—for which the correction factor is equal or close to unity. Therefore, the correction factor was defined as the quotient of the local Reynolds number to the Reynolds number obtained under near-ideal mixing conditions. The correction factor is plotted as functions of the AS inlet Reynolds number in Figure 6, and the maximum correction factor was unity. The average local Reynolds number was calculated for mesh cells in which the energy dissipation rate was 10 times higher than the average in the computational domain, which corresponds to the collision zone area. Therefore, the growth rate is defined as follows:

(20) $$G^{\prime }=\frac{Re}{Re_{id}}G=\zeta \;G,$$

where the correction factor (*ζ*) is calculated using the formula:

(21) $$\zeta =\frac{Re}{Re_{id}}=\frac{\frac{u\cdot \frac{k^{\frac{3}{2}}\;}{\epsilon }}{\nu }}{\frac{u_{id}\cdot \frac{k_{id}^{\frac{3}{2}}\;}{\epsilon_{id}}}{\nu }}=\frac{u\cdot \frac{k^{\frac{3}{2}}\;}{\epsilon }}{u_{id}\cdot \frac{k_{id}^{\frac{3}{2}}\;}{\epsilon_{id}}}.$$

Clearly, the correction factor varies as a function of AS concentration, which corresponds to the mixture viscosity and density at different CA concentrations. The curves indicate the systemic transition flow most likely from the laminar to the turbulent regime. Furthermore, the correction factor is clearly closer to unity for higher flows and Reynolds numbers.

Simulation convergence was obtained after tens of hours (2–3 days) for a single case, that is, modeling of the flow and precipitation process. Such computing times were obtained on AMD Ryzen 3700X CPU unit (using four cores, up to 4.0 GHz).

## 4. Results and Discussion

The modelling results showed that because the different reactor flows depended on the mixture viscosity, the mixing conditions influenced the reaction course. Therefore, the most critical parameters affecting mixing and MoS_2_ precipitation were compared for both reactor types.

**Figure 7:**
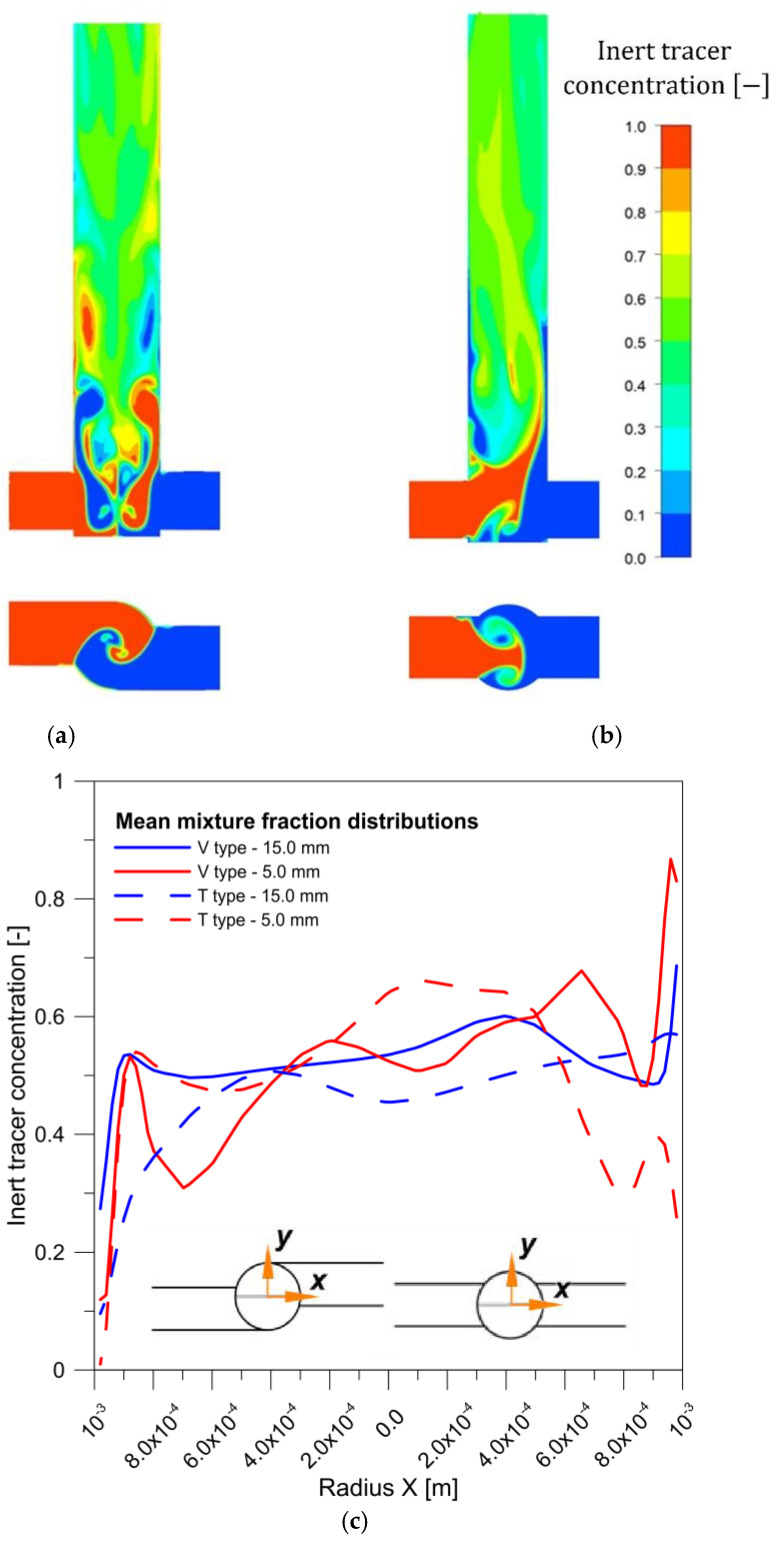
Inert-tracer concentration [−] distribution for 0.6 mol/dm^3^ AS and 20 mL/min flow rate in (**a**) V- and (**b**) T-type reactors, (**c**) comparison of the radial concentration distribution of the inert tracer in a T-type and V-type reactor at two different distances from the reactors bottom.

**Figure 8:**
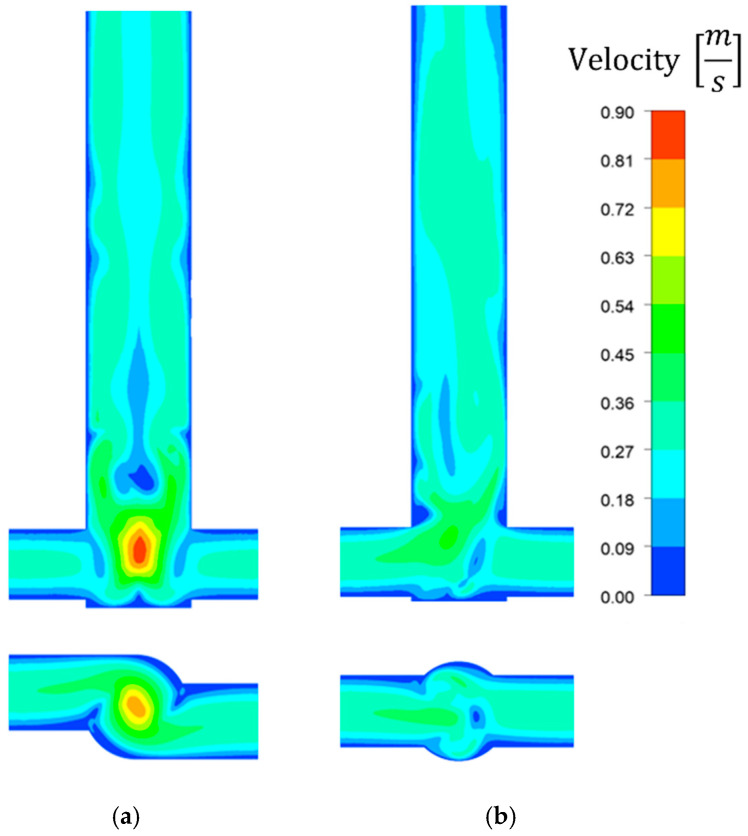
Comparison of velocity [m s^−1^] contour plots for 0.6 mol/dm^3^ AS and 20 mL/min flow rate in (**a**) V- and (**b**) T-type reactors.

Figure 7 shows the influence of the reactor geometry on the inert-tracer concentration distribution, revealing that because of additional axial mixing, the V-type reactor exhibited better mixing conditions than the T-type one. In particular, this can be seen in Figure 7c, which shows the change in concentration of the nonreacting tracer along the radius for two distances from the bottom of the reactor. For the vortex reactor, the concentration is more uniform and closer to the average value than with the T-reactor. Better mixing was achieved at lower substrate concentrations—and, hence, lower mixture viscosities—than at higher concentrations. The velocity profiles shown in Figure 8 are characteristic for the chosen reactor types.

**Figure 9:**
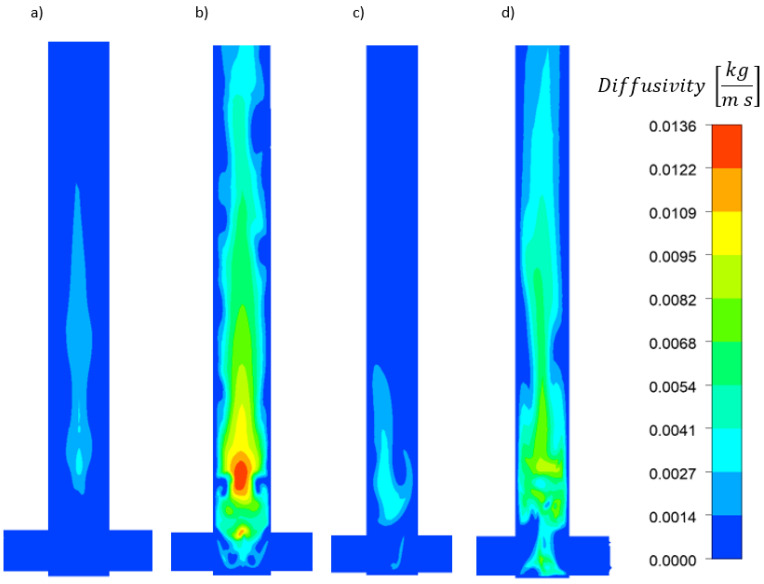
Diffusivity [kg m^−1^ s^−1^] contours for 0.6 mol/dm^3^ AS in V-type reactor at (**a**) 20 and (**b**) 50 mL/min flow rates and in T-type reactor at (**c**) 20 and (**d**) 50 mL/min flow rates.

The diffusivity contour plots show the effect of the high Reynolds number—resulting from the increased flow rate—on increasing diffusivity (Figure 9). Clearly, the increased diffusivity is more pronounced in the V-type reactor and at higher flow rates, and the maximum diffusivity in the T-type reactor is lower than that in the V-type one. The opposite is true for lower flow rates because of the different CA distributions within the reactors. Therefore, the CA concentration critically affects the mixture viscosity and density. At higher CA concentrations, diffusivity decreased because the mixture viscosity increased. Furthermore, the T-type reactor collision axis clearly shifts toward the HMA and CA mixture inlet because the fluids fed into the reactor exhibit different inertias.

**Figure 10:**
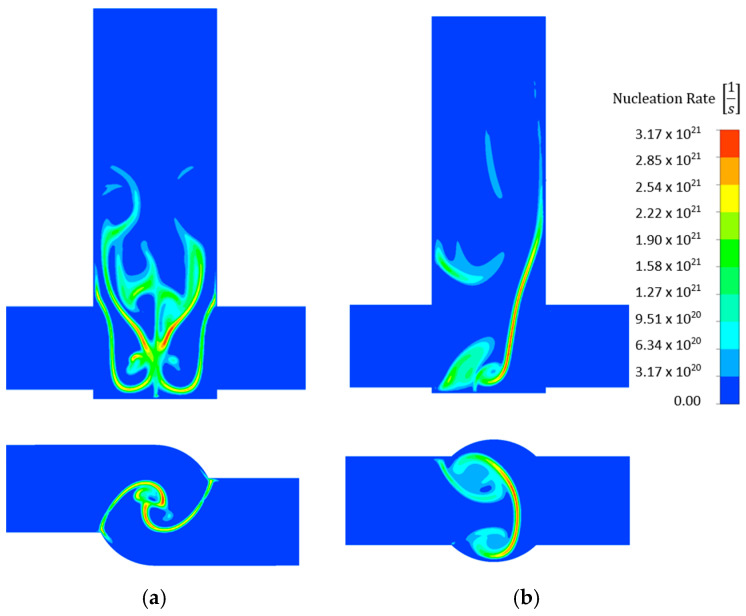
Comparison of nucleation rate [s^−1^] for 0.6 mol/dm^3^ AS and 20 mL/min flow rate in (**a**) V- and (**b**) T-type reactors.

The nucleation rate contours are shown in Figure 10, which compares two types of reactor types at the same flow rate and the same concentrations. Clearly, with increasing AS concentration, the maximum nucleation rate increases over a limited area in the V-type reactor. At the same flow rate and concentration, on the other hand, nucleation occurs over a broader area in T-type than in V-type reactors because less mixing occurs in T-type reactors. Higher nucleation rates were observed at higher flow rates when comparing the same reactor type and concentration.

**Figure 11:**
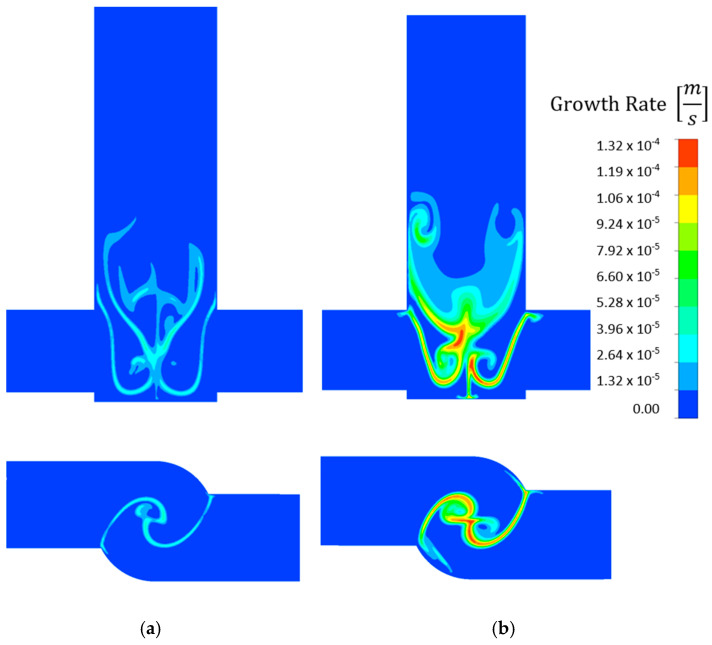
Comparison of growth rate [m s^−1^] contour plots for 0.6 mol/dm^3^ AS and (**a**) 20 and (**b**) 50 mL/min flow rates in a V-type reactor.

**Figure 12:**
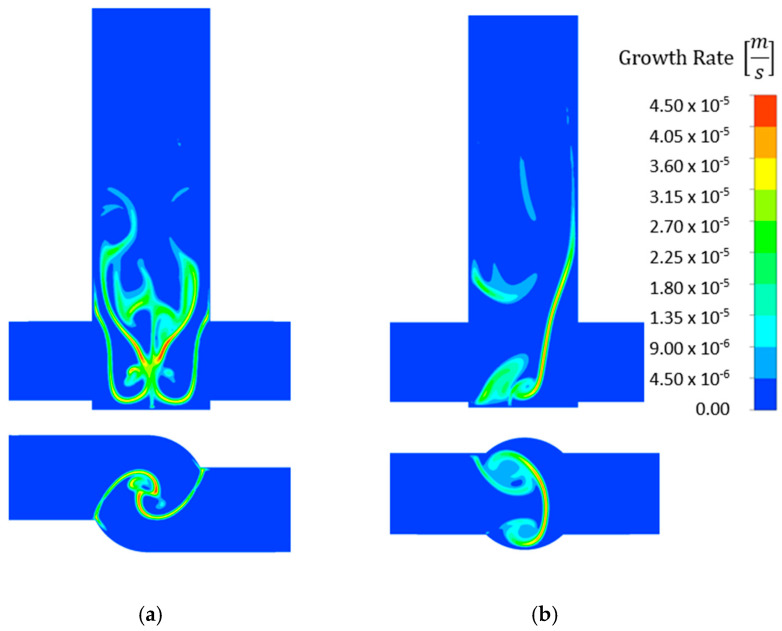
Comparison of growth rate [m s^−1^] contour plots for 0.6 mol/dm^3^ AS and 20 mL/min flow rate in (**a**) V- and (**b**) T-type reactors.

Comparing the same reactor and concentration at different flow rates, considerably higher growth rates were obtained at the 50 mL/min flow rate (Figure 11). Similarly, higher growth rates were obtained in the V-type reactor than in the T-type one (Figure 12) because the reactants were better mixed in the V-type reactor. Like the nucleation rate contour plots, higher growth rates were also observed at higher concentrations. However, the particles grew over a smaller area.

**Figure 13:**
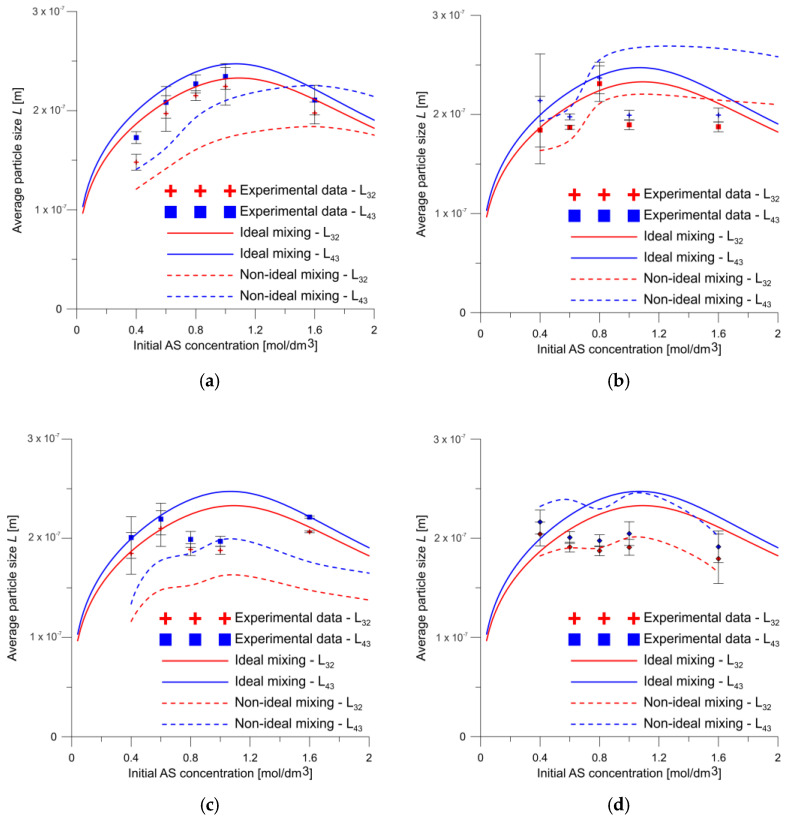
Characteristic particle sizes for V-type reactor at (**a**) 20 and (**b**) 50 mL/min flow rates and for T-type reactor at (**c**) 20 and (**d**) 50 mL/min flow rates (ideal mixing refers to the simulation of a tubular reactor with ideal mixing conditions, and non-ideal mixing refers to CFD modelling that takes into account the reactor geometry and flow conditions).

Although the characteristic particle sizes shown in Figure 13 were in good agreement with those predicted by the ideal mixing model, the fit was slightly worse when the particle size was modelled using CFD. As can be seen, the results of nucleation and growth modelling including the flow field gives a slightly poorer fit than modelling using a pipe reactor model under ideally mixed conditions. However, it allows the effects of changes in geometry and flow conditions on the nucleation and growth process to be taken into account, and therefore, can be used to optimize or scale up the process or study it in different geometries. The model of perfect mixing cannot be used for this purpose, thus its applicability is very limited (or even its not applicable) in real-life engineering applications.

**Figure 14:**
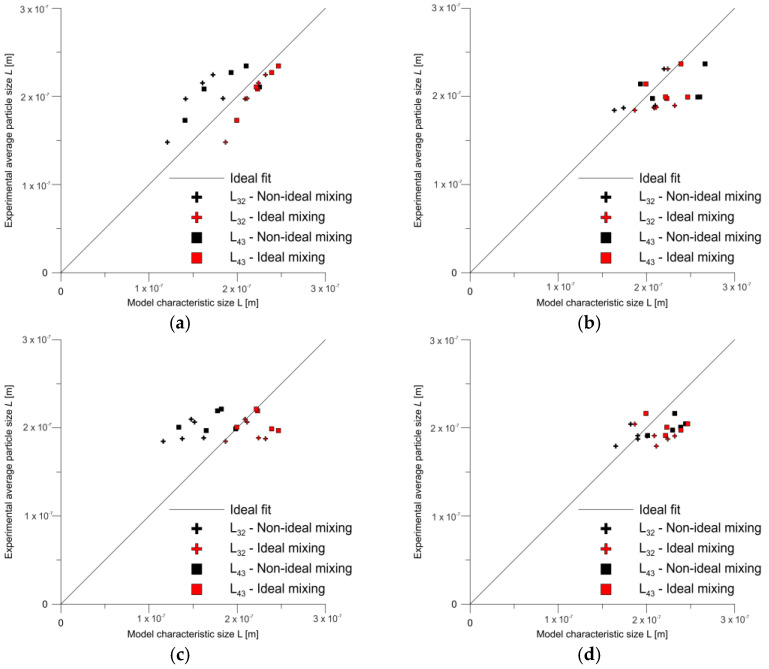
Parity plots for V-type reactor at (**a**) 20 and (**b**) 50 mL/min flow rates and for T-type reactor at (**c**) 20 and (**d**) 50 mL/min flow rates.

Figure 14 shows the parity plots obtained for the results of the tube reactor calculations assuming ideal mixing conditions and the CFD numerical modelling under non-ideal mixing conditions. It can be seen that, regardless of the calculation method, the results are approximately on the perfect fit line. In the case of ideal mixing, the calculations have a clear tendency to overestimate the particle size, whereas in the case of CFD modelling, the average size is undervalued.

**Figure 15:**
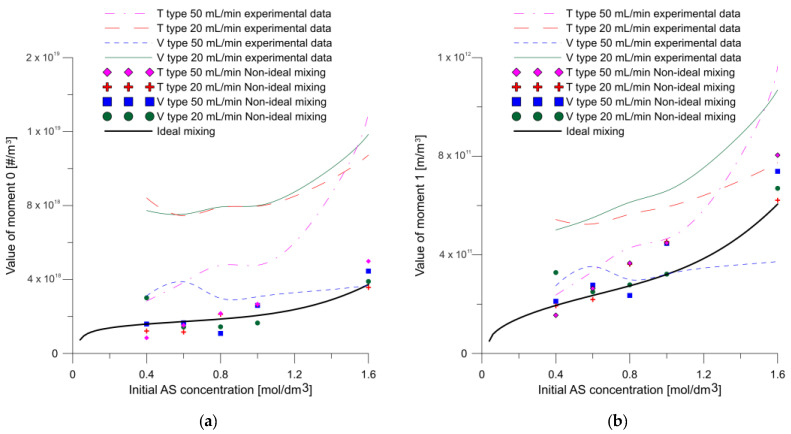

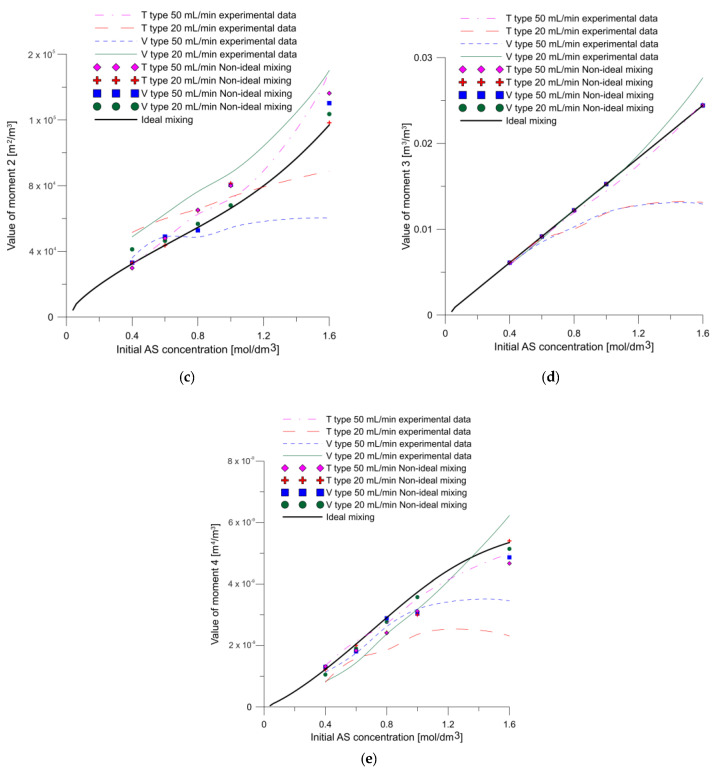
Particle moments in function of initial AS concentration for experimental results and calculated in ideal and nonideal mixing condition (**a**) moment 0, (**b**) moment 1, (**c**) moment 2, (**d**) moment 3, (**e**) moment 4.

Because the determined particle moment distributions show a narrow zero-moment fit (Figure 15), the numerical model exhibits limited ability to accurately predict the number of particles. However, because a better fit was obtained for higher particle moments, the model predicted the resulting particle sizes with good accuracy. Much more accurate numbers of particles were predicted assuming ideal mixing conditions for the model kinetics [24].

The results indicate that the adopted methodology can be successfully applied to predict the precipitation of nanoparticles even in the face of very fast chemical reactions such as the synthesis of metal sulphides. The precipitation of molybdenum sulphide is shown as an example of a very complex process, which is possible to model and predict particle size.

## 5. Conclusions

An improved model of particle nucleation and growth kinetics has been implemented in CFD modelling for MoS_2_ particles synthesized using confined impinging jet reactors. Because the improved model can predict particle size distributions with satisfactory accuracy, it can provide a valuable engineering tool for designing chemical reactors to produce MoS_2_ particles. Parity plots of characteristic particle sizes clearly show that the model can accurately predict particle size distributions regardless of the apparatus geometry.

- The model enables kinetic constants to be determined for other complex chemical reactions, even with limited knowledge of the reaction mechanism, and can be applied with CFD to various reaction processes;
- The effect of the mixing conditions on the chemical reaction was determined using the SST *k*–*ω* model combined with the developed kinetic model to calculate results close to the experimental ones. In addition, the modelling and experimental results deviated more markedly at higher concentrations and lower flow rates, which may be because the reactant mixing was worse and, consequently, deviated even further from the ideal mixing conditions. Under these conditions the viscosity is higher due to the higher concentration of citric acid, what strongly affects the flow parameters, and thus the mixing-limited reaction;
- The concentration and velocity contour plots indicate that the V-type reactor exhibited superior fluid mixing than the T-type one at the same flow rate. However, although this is associated with a more marked pressure drop, the particles were better mixed and nucleated over a much larger area. Fluid mixing is also affected by reagent concentration because fluid viscosity increases with increasing reagent concentration, which affects the Reynolds number.

Future studies may elucidate the reaction mechanism of the wet chemical synthesis of MoS_2_, which will enable the development of a more physical nucleation and growth model. Additionally, particle aggregation, agglomeration, and aggregate and agglomerate disintegration should be investigated in the future to determine their effect on the precipitation process and possible separation of particles from the suspension.

Due to the limited amount of rare earth elements, technologies related to their processing will become increasingly important in the future, making these results important for the production of metal sulphides. Additionally, there are valuable materials with catalytic properties that are likely to find application in the global energy transition.

## Figures and Tables

**Table 1:** Advanced model equations.

$S=\sqrt[{3}]{\frac{7C_{HMA}{\left(1.068\times 10^{-14}C_{AS}\right)}^{2}}{Ks}}$	Supersaturation $(1.068\times 10^{-14}$ = product of sulphide ion equilibrium constants)	(1)
$R_{N_{{\mathrm{MoS}}_{2}}}={a_{ho}}^{\prime }exp\left(\frac{-{b_{ho}}^{\prime }}{\log^{2}\left(S\right)}\right)+{a_{he}}^{\prime }exp\left(\frac{-{b_{he}}^{\prime }}{\log^{2}\left(S\right)}\right)$	Rate of formation from MoS_2_ precipitation	(2)
$R_{N_{S}}=a_{he}C_{AS}{}^{b_{he}}+a_{ho}C_{AS}{}^{b_{ho}}$	Rate of formation from S precipitation	(3)
$G_{{\mathrm{MoS}}_{2}}={k_{r}}^{\prime }S^{k_{D}}$	Linear growth rate coefficient from MoS_2_ precipitation	(4)
$G_{S}=k_{r}C_{AS}{}^{2}$	Linear growth rate coefficient from S precipitation	(5)
$u_{{\mathrm{MoS}}_{2}}=exp\left(\frac{-C_{HMA}}{a}\right)$	MoS_2_ precipitation kinetics share function in model	(6)
$u_{S}=1-exp\left(\frac{-C_{HMA}}{a}\right)$	S precipitation kinetics share function in model	(7)
$R_{N}=u_{{\mathrm{MoS}}_{2}}R_{N_{{\mathrm{MoS}}_{2}}}+u_{S}R_{N_{S}}$	Total formation rate	(8)
$G=u_{{\mathrm{MoS}}_{2}}G_{{\mathrm{MoS}}_{2}}+u_{S}G_{S}$	Total linear growth rate coefficient	(9)
$R_{M}=\frac{\rho }{M}\frac{k_{a}Gm_{2}}{2}$	Substrate consumption rate	(10)
$\frac{d\left(VC_{HMA}\right)}{dt}=-\frac{1}{7}R_{M}$	HMA balance	(11)
$\frac{d\left(VC_{AS}\right)}{dt}=-3R_{M}$	AS balance	(12)
$\frac{dm_{0}}{dt}=R_{N}$	Zero-moment balance	(13)
$\frac{dm_{n}}{dt}=kGm_{n-1}$	Higher-moment balance (for *n* = 1, 2, 3, or 4)	(14)
$\frac{dV}{dt}=0$	Volumetric change (zero for pipe reactor)	(15)

**Table 2:** Constants for the advanced kinetics model.

Constant	Value	Unit
$a_{he}$	5.25 × 10^22^	−
$a_{ho}$	3.97 × 10^23^	−
$b_{he}$	1.89	−
$b_{ho}$	6.58	−
${a_{he}}^{\prime }$	3.05 × 10^15^	−
${a_{ho}}^{\prime }$	1.31 × 10^16^	−
${b_{he}}^{\prime }$	24.04	−
${b_{ho}}^{\prime }$	1.04 × 10^−11^	−
$k_{D}$	0.3533	−
$k_{r}$	0.0102	$m^{5}{\;s}^{-1}{\;\mathrm{mol}}^{-2}$
${k_{r}}^{\prime }$	3.21 × 10^−9^	$m^{5}{\;s}^{-1}{\;\mathrm{mol}}^{-2}$
a	0.034	${\mathrm{mol}\;\mathrm{dm}}^{-3}$

**Table 3:** CA properties at 298.15 K according to Ref. [31].

CA Mass Concentration [−]	CA Dynamic Viscosity [Pa s]	CA Density [kg/m^3^]
0.0000	8.94 × 10^−4^	997.0
0.0643	1.05 × 10^−3^	1022.5
0.0994	1.12 × 10^−3^	1035.3
0.1699	1.28 × 10^−3^	1059.4
0.1982	1.37 × 10^−3^	1068.5
0.2518	1.53 × 10^−3^	1085.0
0.3000	1.69 × 10^−3^	1099.0
0.3400	1.84 × 10^−3^	1110.1
0.3994	2.12 × 10^−3^	1125.7

**Table 4:** Mesh parameters used for different reactor types.

Parameter	V-Type Reactor	T-Type Reactor
Number of cells	2,030,910	2,018,986
Minimum Orthogonal Quality	0.354	0.351
Maximum Aspect Ratio	34.13	33.50

## Data Availability

Data presented in this article is available on request from the corresponding author.

## Sample Availability

Samples of the compounds presented in this article are available from the authors.

## Nomenclature

a	−	share function constant
$a_{he}$	−	heterogenous nucleation rate constant for sulfur particles
$a_{he}^{\prime }$	−	heterogenous nucleation rate constant for MoS_2_ particles
$a_{ho}$	−	homogenous nucleation rate constant for sulfur particles
$a_{ho}^{\prime }$	−	homogenous nucleation rate constant for MoS_2_ particles
$b_{he}$	−	heterogenous nucleation rate constant for sulfur particles
$b_{he}^{\prime }$	−	heterogenous nucleation rate constant for MoS_2_ particles
$b_{ho}$	−	homogenous nucleation rate constant for sulfur particles
$b_{ho}^{\prime }$	−	homogenous nucleation rate constant for MoS_2_ particles
$B_{N}$	$m^{-4}{\;s}^{-1}$	birth rate
$C_{AS}$	${\mathrm{mol}\;m}^{-3}$	ammonium sulphide concentration
$C_{HMA}$	${\mathrm{mol}\;m}^{-3}$	ammonium heptamolybdate concentration
$D_{N}$	$m^{-4}{\;s}^{-1}$	death rate
$F_{1}$	−	auxiliary function in equation for turbulent viscosity in SST *k*–*ω* model
$F_{2}$	−	auxiliary function in equation for turbulent viscosity in SST *k*–*ω* model
G	${m\;s}^{-1}$	total particle growth rate
$G_{{\mathrm{MoS}}_{2}}$	${m\;s}^{-1}$	particle growth rate for MoS_2_ particles
$G_{S}$	${m\;s}^{-1}$	particle growth rate for sulfur particles
k	−	turbulence kinetic energy
$k_{a}$	−	surface shape factor
$k_{r}$	$m^{5}{\;s}^{-1}{\;\mathrm{mol}}^{-2}$	linear growth rate constant for sulfur particles
$k_{r}^{\prime }$	$m^{5}{\;s}^{-1}{\;\mathrm{mol}}^{-2}$	linear growth rate constant for MoS_2_ particles
$K_{sp}$	${\mathrm{mol}}^{3}{\;\mathrm{dm}}^{-3}$	solubility index
L	m	particle size
$L_{10}$	m	characteristic dimension
$L_{32}$	m	characteristic dimension
$L_{30}$	m	characteristic dimension
$L_{43}$	m	characteristic dimension
M	${\mathrm{kg}\;\mathrm{mol}}^{-1}$	molar mass
*m*_0_	$m^{-3}$	moment 0
*m*_1_	${m\;m}^{-3}$	moment 1
*m*_2_	$m^{2}{\;m}^{-3}$	moment 2
*m*_3_	$m^{3}{\;m}^{-3}$	moment 3
*m*_4_	$m^{4}{\;m}^{-3}$	moment 4
n	−	moment index
$R_{M}$	${\mathrm{mol}\;m}^{-3}{\;s}^{-1}$	substrate consumption rate
$R_{N}$	$s^{-1}$	total nucleation rate
$R_{{\mathrm{MoS}}_{2}}$	$s^{-1}$	nucleation rate for MoS_2_ particles
$R_{S}$	$s^{-1}$	nucleation rate for sulfur particles
*S*	−	supersaturation
*t*	s	time
$u_{pi}$	${m\;s}^{-1}$	particle velocity component
$u_{{\mathrm{MoS}}_{2}}$	−	MoS_2_ precipitation share function
$u_{S}$	−	S precipitation share function
V	$m^{3}$	volume
x, y, z	m	Cartesian coordinates
Greek symbols		
ρ	${\mathrm{kg}\;m}^{-3}$	density
ζ	−	share function
